# Vaccination against parasites – status quo and the way forward

**DOI:** 10.1186/s40813-016-0047-9

**Published:** 2016-12-15

**Authors:** Anja Joachim

**Affiliations:** Institute of Parasitology, University of Veterinary Medicine Vienna, Veterinaerplatz 1, A-1210 Wien, Austria

**Keywords:** Swine, Vaccine, Immunity, Nematodes, Protozoa

## Abstract

Although vaccination against various pathogens is integral to health management of swine, vaccines against parasites have not yet been commercialized for the use in pigs. The incentive to develop and commercialize anti-parasitic vaccines in swine are twofold; on the one hand parasitic diseases which are economically important, such as ascarosis and neonatal coccidiosis, could be controlled in a sustainable manner; on the other hand, the transmission of zoonotic parasites, such as *Toxoplasma gondii* or Cysticercus cellulosae, could be effectively interrupted. Although experimental research indicates that vaccination against a number of porcine parasites is feasible, development and commercialization of potential vaccines so far has been very slow, as our knowledge on the host-parasite interplay in porcine parasitic infections is still very limited. In the light of growing concerns regarding consumer health and antiparasitic drug resistance, however, it is timely to re-direct R&D efforts to the development of biological control options.

## Background - vaccines against parasites

In modern swine medicine, vaccination against various pathogens is an integral part of the health management. However, currently not a single vaccine against parasites of swine is commercially available. Compared to viral and bacterial pathogens, there is a general scarcity for anti-parasite vaccines; only two anti-nematode vaccines, one anti-tick-vaccine and a handful of antiprotozoal vaccines are available for domestic animals. The reasons for such a limited number of anti-parasite vaccines are manifold. For many parasitic infections the development of immunity is slow, and especially in livestock animals, the required time is too short for the vaccine to be of value before animals go to slaughter.

Since parasites, especially helminths, are prime manipulators of the immune system, immunity is often also incomplete and not sufficient to interrupt the life cycle, which aids in the continuation of transmission in a population regardless of vaccination. For host species with a fast turnover the development of anti-parasite vaccines are considered too expensive and especially in intensive pig (or poultry) production chemical control is cheaper, easier to apply and is considered to have a broader market. The only anti-parasite vaccine currently available for use in pigs, the anti-Cysticercus cellulosae-vaccine for the prevention of porcine cysticercosis (which leads to infection of humans with the tapeworm *Taenia solium*), is not yet commercially available although it has a very good efficacy [19]. Simplified and cost-effective application will have to be developed to apply this highly effective vaccine to pigs on a large scale, such as the expression of the recombinant antigen in feed plants [28].

However, the development of vaccines against parasites is still a significant research topic in medical and veterinary sciences. Pathogenic and therefore economically important diseases, especially those which are insufficiently controlled by available chemotherapeutics or have developed resistance against them that cannot be immediately overcome, are still in the focus. In addition, zoonotic parasites represent an attractive target under the One Health aspect, and finally there is a growing public interest in organic production of food free of chemicals, which is fostered by consumer concern about drug residues in meat, eggs or milk.

### Which swine parasites are to be considered for vaccines?

The only group of vaccines against parasites that is well developed are the anti-coccidial vaccines for poultry, i.e. *Eimeria* in chicken and turkey. Live virulent and attenuated vaccines are the predominant types on the market; some strains have been used for more than 50 year without significant alterations [39]. Technically, vaccination with life parasites, in this case *Eimeria* oocysts from several relevant species, represents an infection of susceptible animals under controlled conditions. The parasites undergo the complete life cycle and recirculation of oocysts induces a natural booster, rendering chicken immune after several cycles of reproduction. It is assumed that vaccine strains which are susceptible to anticoccidials can displace resistant field isolates when applied repeatedly [5].

In order to be a candidate for vaccine development, parasites have to fulfil several prerequisites. They must be sufficiently pathogenic to induce disease and or/economic losses that can be ameliorated by vaccination, and natural infections must be immunogenic and induce protective immunity and an immunological memory. Amongst the most common swine parasites, some fulfil these criteria. *Sarcoptes scabiei* var. suis, the mange mite of pigs, causes severe economic losses and frequently serious disease in pigs when untreated [6]. Currently, control of porcine sarcoptic mange relies on the application of acaricides and the maintenance of mite-free herds [18]. Immunity against scabies has been described in different species including humans [43]. Vaccination has been attempted in rodent models [11] and other species, and it might also be feasible in pigs.

*Strongyloides ransomi* is a nematode which is most commonly transmitted with the colostrum after reactivation of hypobiotic larvae in the sow. It causes transient diarrhoea in suckling piglets and induces strong immunity in the adult intestinal stage which leads to rapid expulsion by the host. The immune mechanisms of expulsion have been investigated for other *Strongyloides* species [51], therefore this nematode also fulfils the principle criteria for a vaccination candidate. This is also true for other nematode species of swine that are expelled by action of the gut immune system in pigs, the large roundworm, *Ascaris suum* [24], and the whipworm, *Trichuris suis*. In contrast to these the nodule worm *Oesophagostomum* induces only a weak reaction of the host’s immune system [1], making the latter unsuitable for immunological intervention. With regard to the economic importance, porcine nematodes, especially *Strongyloides*, *Trichuris* and the nodule worms, have decreased in prevalence sind the advent of broad-spectrum anthelminthics and modern management, although *A. suum* prevalences can still be considerable, especially in traditional management systems or on organic farms [16, 29–31].

Of the protozoa, *Toxoplasma gondii* is an attractive candidate for vaccine development, as it is the most important foodborne zoonotic parasite on a global scale, and interruption of the life cycle by preventing cyst formation in animals used for meat production would effectively truncate foodborne transmission. A range of promising vaccine designs and candidates has been used in mice [20], and also in pigs (e.g. [3] for recent works). While it is assumed that vaccination of livestock against *Toxoplasma* can prevent infection in humans, the infection in pigs causes only minor production losses or animal health problems and the attractiveness of such a vaccine for pig producers is certainly only limited unless the label “*Toxoplasma*-free pork” has economic advantages. In suckling piglets *Cystoisospora suis* (syn. *Isospora suis*) causes intestinal infections which may cause transient diarrhoea mostly in the second week of life. Due to the peculiarities of the porcine neonatal immune system (see below) and the strong age resistance to *C. suis* in piglets older than three weeks [46, 47], it is currently assumed that only pigs older than six weeks can mount an appropriate immune response. For this and other reasons vaccination of piglets against *C. suis* is not considered feasible. However, alternative approaches have recently been evaluated (see below).

### Parasite control in swine production – current status

Currently, antiparasitic treatment schemes for pigs is comprised of a “standard” application scheme for different production branches; they are not “tailor-made” or risk-based and not driven by diagnosis, since metaphylactic application of antiparasitic drugs during the prepatent phase (when parasites cannot readily be detected by routine screening) is preferred to prevent dissemination of environmental stages (especially nematode eggs or coccidia oocysts) and infection of the litter or herd. Complete elimination from a herd is often difficult to achieve due to high prevalences, frequent distribution and durable environmental stages, the best example being eggs of *A. suum* which are almost impossible to inactivate and which can remain infectious for years under suitable conditions [27, 45]. An exception is the control of mange; *S. scabiei* has no long-lived environmental stage and relies on direct contact for transmission, so systematic application of acaricides can effectively reduce infection and stamping out the parasite on a farm is possible when quarantine measures and proper diagnostic screening are in place (e.g. [38]). However, sustainability is jeopardized by the development of acaricide resistance as reported from human scabies [22]. Integrated measures like complete all-in-all-out and disinfection with effective chemicals can relieve the infection pressure of endoparasite infections [15] but eradication is generally considered not feasible. Although resistance against anthelmintics in pig nematodes seems to be restricted to *Oesophagostomum* at low frequencies [2, 9, 41] and resistance to anticoccidials in the control of *C. suis* is currently not reported, the limited number of substances available especially for parasite control in pigs is of concern; especially because no routine tests are available for the detection of parasiticide resistance, and no programs to delay the development of resistance (like shuttle programs as for chicken coccidiosis prevention [44] or equine cyathostominosis) are in place. Alternative control strategies (for review see [32]) haven been shown to be effective but are currently not commercially available. It must therefore be assumed that, although currently parasites may not be considered as an issue of major concern in pig health and production, in the long run alternatives to the current chemotherapy must be sought to maintain appropriate control and efficacy of available drugs.

### Consequences of neonatal enteric infections: parasites and their buddies

At the time of birth the porcine immune system is poorly developed (for review see [47]); intestinal Peyer’s patches contain almost no immune cells and the gut epithelium and subepithelial tissues are only completely populated with T- and B-cells and antigen-presenting cells at about six weeks of age, leaving ample time for pathogens to establish and reproduce. At the same time, the gut microbiota are establishing and infections with pathogens at a very early age may have a number of consequences beyond transient parasite infection. Synergistic effects have been described for *C. suis* and toxigenic *Clostridium perfringens* where timely anticoccidial treatment also alleviated the effects of clostridiosis [26], showing that *C. suis* infections promote adhesion of clostridia to the intestinal mucosa, exacerbating the effects of bacterial infection. Preliminary studies also indicated that infections with *C. suis* alter the succession of bacterial communities in neonatal pig gut, delaying the establishment of lactobacilli (as reviewed in [37]). As interactions between microbiota and the immune system are key to the development of a functional immune system [40] such events may have lasting effects on the development of intestinal and immune functions. As such alterations have been described for other intestinal parasitic infections [21], a role of intestinal parasitic infections in gut health should be re-evaluated in pigs, too.

### *Cystoisospora suis* - a candidate for vaccination?

As mentioned above, *C. suis* is an important cause of neonatal diarrhoea; infected animals excrete several million oocysts in the patent phase of infection and the dissemination within and between litters accounts for a rapid spread of the parasite with the consequence of transmission to the majority of piglets within the first week after birth. Although infections are transient with creamy to watery non-haemorrhagic diarrhoea for one to six days, affected animals often develop poorly and stay smaller even until weaning compared to healthy (treated) litter mates [25], which accounts for the financial losses attributed to this disease [17, 33] requiring treatment. In addition, dysbiosis may contribute to increased morbidity (see above) and require antibiotic treatment [7]. Good control of oocyst excretion and coccidiosis-related diarrhoea is achieved by metaphylactic treatment of piglets on the third to fifth day of life with a single dose of toltrazuril (20 mg/kg of body weight) but recently questions about the sustainability of “blanket treatment” of piglets in terms of resistance and drug residues in meat have risen (see [37]), and a call for alterative control strategies has been voiced.

*C. suis* as a member of the Apicomplexa, which have a strictly intracellular development in the host, was assumed to be under the control of the cellular immune system, mainly NK cells, CD4^+^ and CD8^+^ T-cells, while antibodies are probably not protective [47]. Phenotyping of cells after primary infection revealed that several subpopulations of T-cells (especially γδ-T cells and, T_H_ cells) were decreased in peripheral organs (blood, spleen) but increased in the jejunum upon infection), and after challenge infection (5 months after primary infection) these cell populations also produced interferon-γ (which is crucial for the defence against apicomplexan parasites) and were able to proliferate upon antigen stimulation [8, 48, 49]. Thus, despite considerable individual reactions, it can be assumed that *C. suis* can induce specific primary and adaptive immune reactions in pigs including the induction of an immunological memory.

Since a relative of *C. suis, Cryptosporidium parvum* which also inhabits the epithelium of the small intestine, is at least partially controlled by specific antibodies [23], investigations in the possible role of anti-*C. suis* antibodies were made and colostral transfer of antibodies resulting in high serum levels in piglets was described, and IgA levels in the blood of piglets experimentally infected with *C. suis* soon after birth were negatively correlated with diarrhoea [34]. When sows were inoculated before birth with high doses of *C. suis* oocysts, no clinical signs or oocyst excretion were noticed but the levels of immunoglobulins (especially IgA) in their blood, colostrum and milk were correlated with a decrease in diarrhoea and oocyst excretion in their experimentally infected off spring compared to piglets from non-superinfected sows [35], indicating that application of oocysts to sows ante partum can confer at least partial protection against *C. suis* in piglets. It was also concluded that the role of the sow in spreading the parasite is probably minor since even after infection with high doses (100,000 oocysts / sow) no shedding was observed; however in the immunity against *C. suis* the role of the mother in the provision of colostrum containing protective substances is probably pivotal. From this it is currently assumed that immunological control of neonatal porcine cystoisosporosis would have to be conferred as a maternal vaccination which could be applied in time before the infection of the new-born piglets to immunologically mature gilts or sows and could be boostered by natural infections circulating in a herd.

### Determination of vaccine candidates – the way forward

Current vaccines against coccidian in chicken are mostly virulent or attenuated live vaccines (see above). This technology requires the use of animals for production of vaccines with all its disadvantages (biosafety, ethical concerns etc.). Lately, a subunit vaccine against *Eimeria maxima* of chicken was developed and marketed for use in maternal immunization [36, 42]. Although it is also produced in animals and its lasting success in the field still remains to be evaluated, the concept of inactivated vaccines against parasites has received a significant incentive with this development. Until recently, the search for new vaccine candidate molecules was slow and cumbersome due to the lack of cost-effective high yield / high throughput *in vitro* techniques for parasite propagation and screening. New techniques in biotechnology and bioinformatics have enabled rapid and cost-effective screening of genomes and transcriptomes of parasites for vaccine and drug target candidates (as reviewed in [4, 12], and others) including *A. suum* [13]*, T. suis* [14]*, T. gondii* (www.toxodb.org/) and *C. suis* (Palmieri, submitted). For *C. suis* a genome size of 83 Megabases encoding >8300 genes is estimated, and a recently developed pipeline for the search of vaccine candidates in apicomplexan parasites (Vacceed; [10]) has detected 562 candidates in *C. suis* which now need to be evaluated further *in silico, in vitro* (using a cell culture system supporting the complete life cycle of *C. suis*; [50]) and *in vivo*.

## Conclusion

In summary, although vaccines against porcine parasites do not seem to be an immediate issue for pig industry and health, the time to get started has never been better, as new tools and technologies are greatly accelerating the achievements in this field of veterinary medicine.

*C. suis* would be an attractive candidate for vaccine development, as preliminary data have shown that a protective effect could be achieved by immunisation of sows; however, more basic and applied research will be needed to fully understand the mechanisms that lead to protection.

Joint forces of the veterinary profession, immunology, bioinformatics and biotechnology specialists will be required to develop and test new vaccine concepts and vaccines to be suitable for a competitive market. Combinations of vaccines and optimised delivery systems will have to be developed alongside to incorporate anti-parasite vaccines into the health management systems of pig production.

## Abbreviations

*A. suum*: *Ascaris suum*
*C. suis*: *Cystoisospora suis*
*S. scabiei*: *Sarcoptes scabiei*
*T. gondii*: *Toxoplasma gondii*
